# Real-time recognition of spraying area for UAV sprayers using a deep learning approach

**DOI:** 10.1371/journal.pone.0249436

**Published:** 2021-04-01

**Authors:** Shahbaz Khan, Muhammad Tufail, Muhammad Tahir Khan, Zubair Ahmad Khan, Javaid Iqbal, Arsalan Wasim

**Affiliations:** 1 Department of Mechatronics Engineering, University of Engineering & Technology, Peshawar, Pakistan; 2 Advanced Robotics and Automation Laboratory, National Center of Robotics and Automation (NCRA), Rawalpindi, Pakistan; 3 College of Electrical & Mechanical Engineering (CEME), National University of Sciences and Technology (NUST), Islamabad, Pakistan; 4 Department of Electrical Engineering, Hitec University, Taxila, Pakistan; Ministry of Natural Resources North Sea Bureau, CHINA

## Abstract

Agricultural production is vital for the stability of the country’s economy. Controlling weed infestation through agrochemicals is necessary for increasing crop productivity. However, its excessive use has severe repercussions on the environment (damaging the ecosystem) and the human operators exposed to it. The use of Unmanned Aerial Vehicles (UAVs) has been proposed by several authors in the literature for performing the desired spraying and is considered safer and more precise than the conventional methods. Therefore, the study’s objective was to develop an accurate real-time recognition system of spraying areas for UAVs, which is of utmost importance for UAV-based sprayers. A two-step target recognition system was developed by using deep learning for the images collected from a UAV. Agriculture cropland of coriander was considered for building a classifier for recognizing spraying areas. The developed deep learning system achieved an average F1 score of 0.955, while the classifier recognition average computation time was 3.68 ms. The developed deep learning system can be deployed in real-time to UAV-based sprayers for accurate spraying.

## Introduction

Agriculture is recognized as the largest sector in ’Pakistan’s economy. It contributes to about 24% of GDP (Gross domestic product). Additionally, it is the largest foreign exchange source and feeds the entire urban and rural population [1]. In Pakistan, nearly 62% of the country’s population dwells in rural areas and depends directly or indirectly on agriculture for their livelihood [2]. Pakistan has fallen behind since the 1980s in agriculture due to the lack of technology being used to overcome the losses. These losses are caused by pests and insects, which ultimately reduces productivity. Agrochemicals are used to overcome the aforementioned issue, though if sprayed manually in the crop field, they can severely impact a typical person’s life. Furthermore, the overuse of pesticides has ramifications on human health. According to the World Health Organization (WHO), one million adverse effects were reported when manual spraying of pesticides is employed in the crop field [3]. Children are specifically susceptible to the harmful impacts of agrochemicals, and even very little exposure during the development of a child can harm their health [1]. Remedial actions were needed to be taken to safeguard the population against these effects, and taking advantage of the new technologies led to the introduction of Unmanned Aerial Vehicles (UAVs) and other kinds of robots in this field. UAVs have been used in different precision agriculture applications such as spraying [4, 5], detecting weeds [6–8], disease detection [9–11], etc. Among these applications, the spraying operations need to be robust, i.e., to avoid spraying in areas where there are no crops, as the payload capacity of a UAV is minimal. The ability to accurately recognize spraying areas (crops and orchards) becomes more vital in autonomous UAV based spraying systems. The latest advancement in deep learning and the internet of things (IoT) can help significantly in developing efficient autonomous systems [12–15]. The study aims to extend this by developing a deep learning-based real-time robust recognition system for the UAV to recognize the spraying area for precision spraying.

The remaining paper is organized as follows: Section 2 discusses the related work. The proposed methodology is presented in section 3. Experiments and results are described in sections 4 and 5, respectively. Section 6 discusses the results, while Section 7 concludes the article.

## Related work

’UAVs’ are already established in different fields [16, 17], and it is expected that its market will increase to $200 billion in the upcoming years [18]. Yamaha developed its first model (Yamaha RMAX) for crop monitoring and pest control, whose production was discontinued in 2007 [3]. Y. Huang *et al*. [19] developed a spray system for the UAV application platform. The integration of the spray system with the UAV resulted in an autonomous spray system that was used for pest management and vector control. A Pulse Width Modulation (PWM) controller for UAV precision agriculture sprayer was employed [20], and the UAV was remotely controlled or flown autonomously by preprogrammed flight plans. The PWM controlled technique provided higher precision for spraying applications. A low-volume sprayer was developed for vector control and extendable to crop production management [21]. The system was able to deliver liquid to 30m swath width, 42m downwind. The technology was found useful for providing chemicals precisely to the right place at the right time. According to Bruno S. Faiçal *et al*. [22], an architecture was proposed for UAV having a wireless sensor network (WSN) for pesticide spraying in the crop fields. The proposed architecture reduced the risk of errors caused by adverse weather conditions. WSN provided feedback on pesticide concentrations, based on which route was changed gradually until the node identified the product’s proper application. In another instance, a spray system was mounted on an unmanned aircraft [23]. The system was deployed in high-value specialty crops in California. The system had a UAV and an associated ground control station providing remote piloting of the aircraft. Spraying application rates and deposition rates were comparable to the manned observed aerial spraying. External problems like wind speed changes and direction required for spraying on crop fields were addressed [24]. An artificial neural network was proposed on programmable UAVs. The UAV was programmed to spray chemicals on the target crop based on a dynamic context. Particle Swarm Optimization (PSO) was employed for finding optimum parameters on which neural network was trained for improving the UAV route in dynamic environments. Results showed an improvement in precision spraying in dynamic environments by using the proposed technique [24]. Xinyu Xue *et al*. designed a UAV-based automatic control spraying system. It had a single-chip microcomputer with an independent functional module, which allowed route planning software for directing the UAV to the desired spray area. The UAV flew in the designated spray routes with precision [25]. In another instance, a quadcopter (FREYER) was developed, which carried pesticides spraying the farm. To reduce the ’farmer’s work, a user-friendly interface for the farmers was developed. The drone’s control was performed through an android app using a Wi-Fi module that was interfaced in the drone [26]. Similarly, a drone-mounted sprayer was developed and evaluated for pesticide applications of crops by Yallappa *et al*. the entire drone-mounted operation sprayer was controlled through a transmitter at the ground, and a live spraying operation was monitored using a first-person view (FPV) camera. The sprayer was useful in places where human interventions were not possible and helped reduce the cost of pesticide application and environmental pollution [27]. Likewise, a vision-based autonomous spray system was developed by B. Dai *et al*., to design a UAV system for autonomous completion of a precise spraying mission in an unsupervised manner [28]. During the mission ten foam boards were numbered randomly and fixed on a vertical wall and were assumed as fruit areas that were needed to be sprayed preciously; the system included the design, algorithm for task scheduling, and used vision for identification and localization which performed efficiently [28]. Sheng Wen *et al*. [29] developed a variable spray system using neural network-based decision making. Back Propagation (BP) neural network model was trained based on the factors affecting droplet deposition. The factors were ambient temperature, humidity, wind speed, flight speed, flight altitude, propeller and nozzle pitch, and the prescription value. The BP neural network was combined with variable rate spray control with multiple sensors collecting real-time information. The spray system’s flow rate was regulated for determining the deposition rate based on the predicted deposition amount neural network [29].

It is evident from the literature that extensive work has been carried out to perform spraying operations through UAVs, but the primary focus remained on the task of spraying instead of onboard recognition systems, which is of utmost importance in spot spraying applications. It is estimated that only 50% of targets are being sprayed through UAVs when the altitude is less than 1m [30], which makes it even more important for such devices that a system for accurate recognition is in place. Pengbo Gao *et al* [31] developed a recognition system for crops and orchards for UAVs using a Mutual Subspace Method (MSM). However, the system was able to achieve only 65.1% accuracy for real-time recognition of crops. This research aimed to develop a more accurate recognition system for crops using a deep learning approach to view the computational constraints associated with the UAVs. It is assumed that low computation shape and color detection systems employed with a less complex deep learning model can achieve the aforementioned objectives.

## Methodology

**Fig 1:**
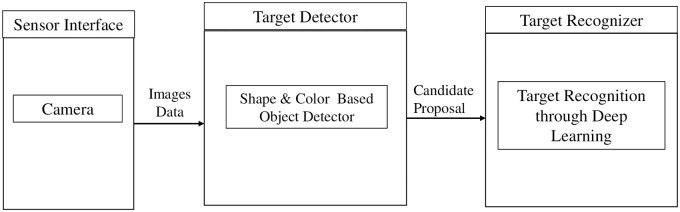
Proposed target recognition framework.

The proposed framework aims to accurately recognize the target by recognizing and locating the target within an image plane. The proposed framework comprises two steps to accomplish the goal, as shown in Fig 1. In contrast to conventional techniques, for recognizing targets through predefined knowledge of target such as shape, texture, etc., which are prone to errors, the proposed framework primarily depends upon a deep learning classifier for target recognition. The two steps that constitute the proposed methodology are explained in the subsequent section.

### Target proposal

This component of the framework considers the computational constraint associated with the UAV and is responsible for generating the candidate proposal for the target recognizer module. This component uses shape and color detection algorithms to enhance the effectiveness of the posterior recognition stage. Contours are utilized to detect shapes, while blue-green red (BGR) color space is used to detect color. Based on the color and shape information, this component can be utilized by the posterior stage for computing the target’s relative pose and ultimately used for readjusting the UAV. Furthermore, this combination of shape and color can provide an intraclass classification of the targets (e.g., spraying area, non-spraying area, plants, etc.).

### Target recognizer

This module’s primary goal is to accurately detect targets and minimize the errors linked with target detectors employing predefined knowledge of color, shape, etc. The target recognizer module involves two steps: Off-board and onboard/Real-time recognition systems briefly explained as follows.

### Off-board recognition system

**Fig 2:**
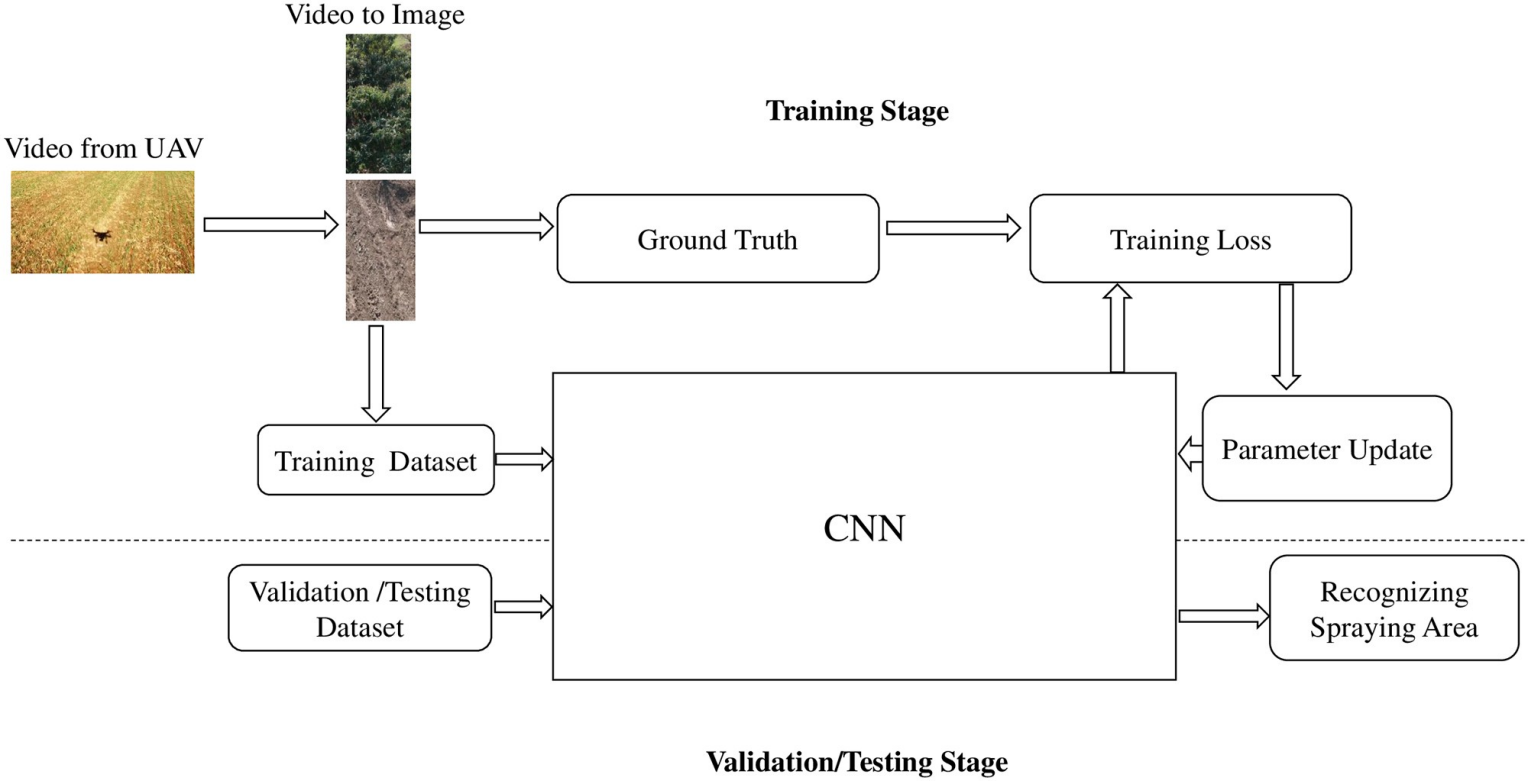
Off-board recognition system.

It is used for training and then validating the trained system which is used in the online recognition system for real-time target detection. The module comprises two stages: The training and Testing/Validation stage. During the preprocessing stages, videos are converted into images through the Joint Photographic Experts Group (JPEG) converter. The images are divided into two datasets for training and testing the classifier [32, 33]. The training process is continued until the loss value is less than 0.1, while the testing/validation dataset is mapped into prediction class by the classifier, as illustrated in Fig 2. The training data set is labeled manually, while TensorFlow and Keras open-source deep learning are employed for experimentation.

The experimental platform used is the Intel i7 7700HQ quad processor, 16 GB RAM, and NVIDIA GeForce GTX 1080 GPU.

### On-board/real-time recognition system

**Fig 3:**
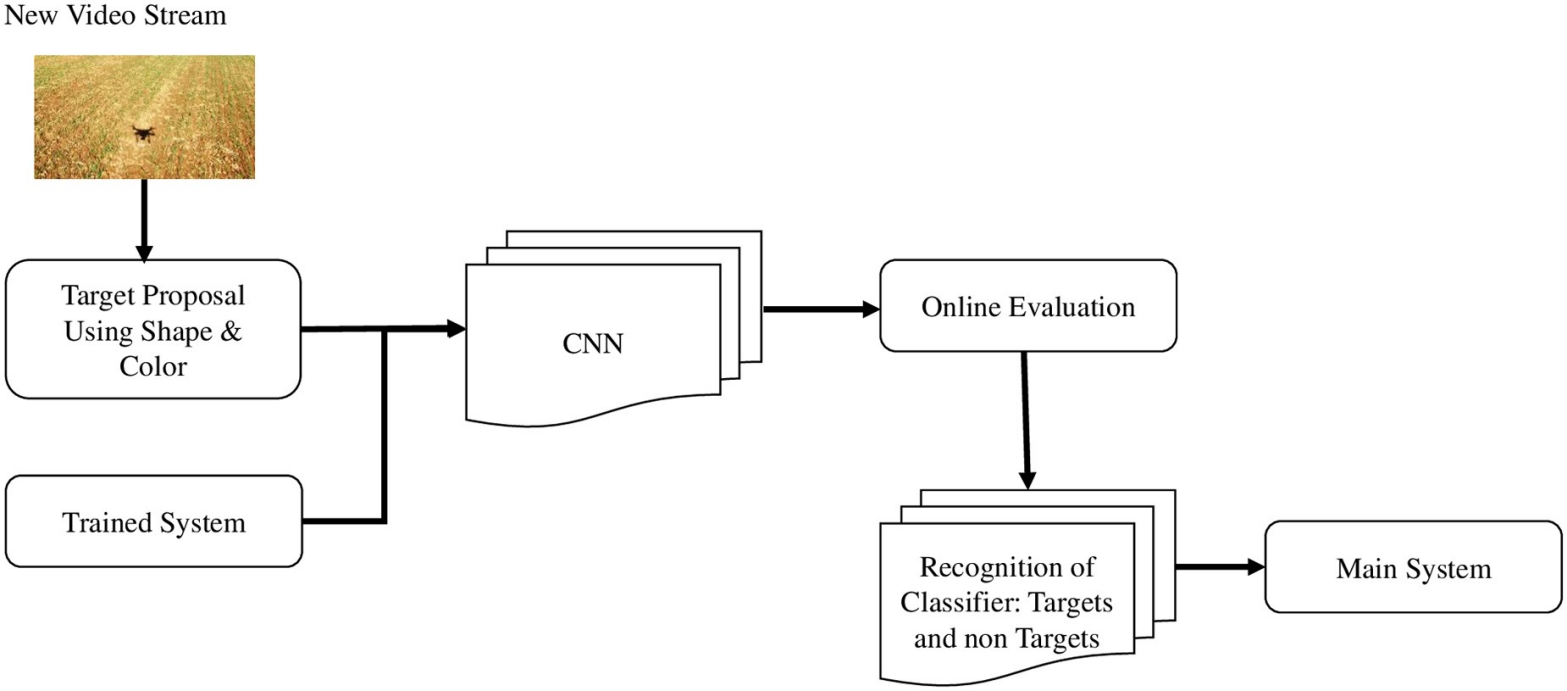
On-board/real-time recognition system.

This module is deployed as the primary target recognition algorithm in real-time after image processing is performed through the bottom camera. During the recognition system, a new video is captured, the target proposal component proposes candidates, and through the trained system and supervised learning classifier, the system recognizes the target in real-time, as depicted in Fig 3.

Different supervised learning classifiers are implemented for training and evaluation, the details of which are provided in the experiment section. After evaluating experiments, the selected classifier architecture consists of five (05) Convolutional layers, four (04) max-pooling layers, five (05) dropout layers, two (02) dense layers with one (01) fully connected layer having a hidden layer of 512 units. The last layer is a dense layer with sigmoid activation. The size of the filter is 3*3.

## Experimentation

To validate the robustness of the developed system, simulated and real flight experiments were conducted. The experiment’s main aim was to test the reliability of the system by repeating the experiment multiple times. Five custom Convolutional Neural Network (CNN) models (Table 1) by varying the layers were considered during the study for evaluation.

**Table 1:** CNN configurations.

Architecture	No. of Conv Layers	No. of Max Pool Layers	No. of Dropout layers	Filter Size	Feature Map
CNN1	3	2	2	3*3	32,32,64
CNN2	4	3	3	3*3	32,32,64,128
CNN3	5	4	5	3*3	32,32,64,128,128
CNN4	6	5	4	3*3	32,32,64,128,128,256
CNN5	7	6	5	3*3	32,32,64,128,128,256,256

The subsequent sections describe the simulation experiments and field experiments in detail.

### Experimental scenario

**Fig 4:**
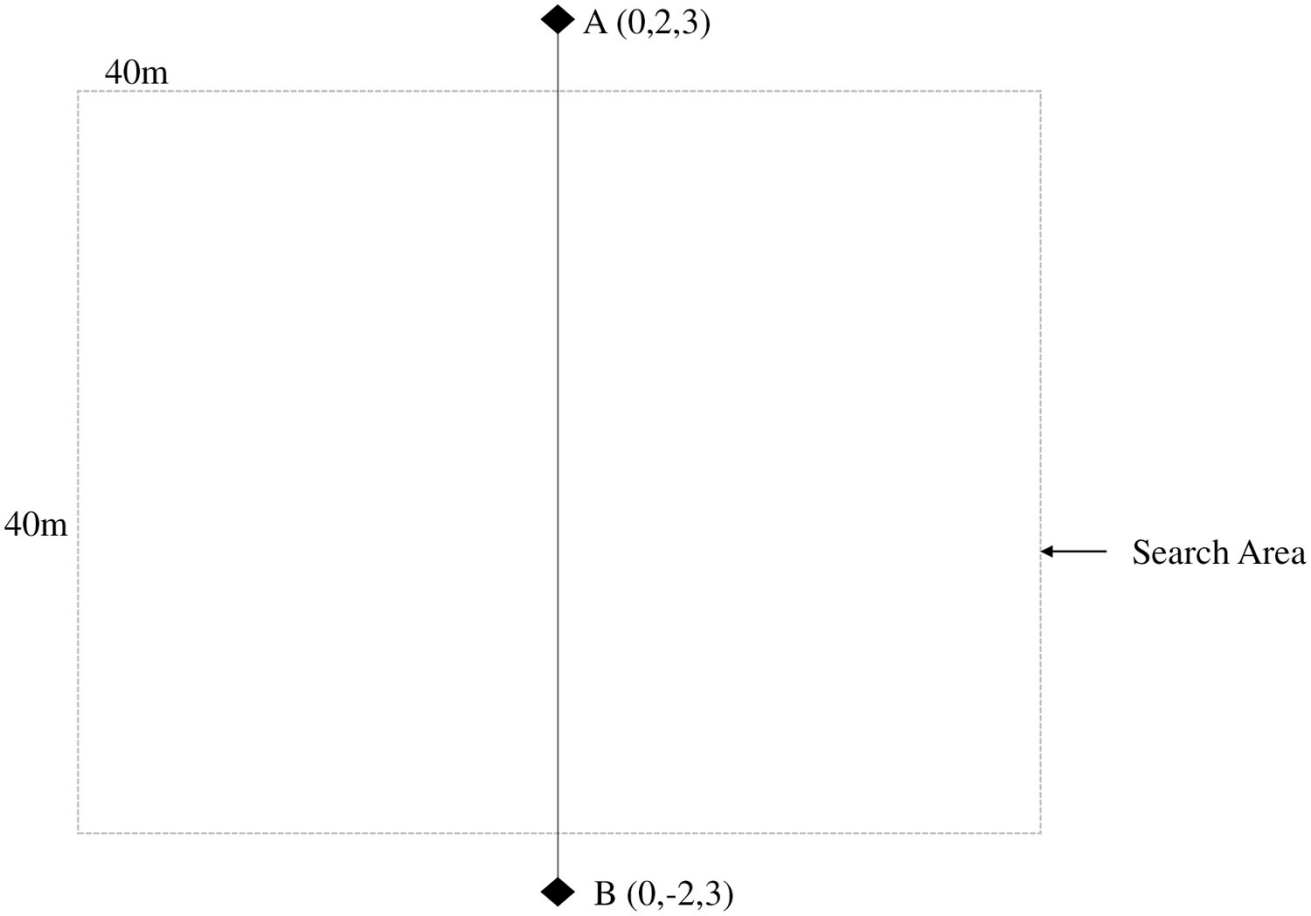
Experimental scenario. A and B represent waypoints while the search area was 40m*40m.

The experimental scenario for both the simulation and field tests is shown in Fig 4. Two-way points A and B were selected, and the UAV had to take off and move from one waypoint to another with a height of 3m. The targets were placed in between the waypoints.

### Simulation experiments

It is essential to refine and test the system before deploying the system on any field flight test. Hence, different simulation approaches were explored in the study to obtain realistic results. PX4 software in loop (SITL) was chosen for simulating the experiments because its simulation is almost ideal. Gazebo robotic simulator [34] and autopilot software stack was used for setting up the environment. The simulated world and the quadcopter model were developed similar to the real experimental world. The environment was developed using a single row of crops immersed in a muddy background identical to the real field. Crops were considered targets with shape and color (light green) similar to standard crops and mud. A simulated camera model similar to the actual camera’s parameters was used in the real tests attached to the quadcopter. The PX4 parameters were adjusted accordingly to make the flying velocity similar to field tests.

**Fig 5:**
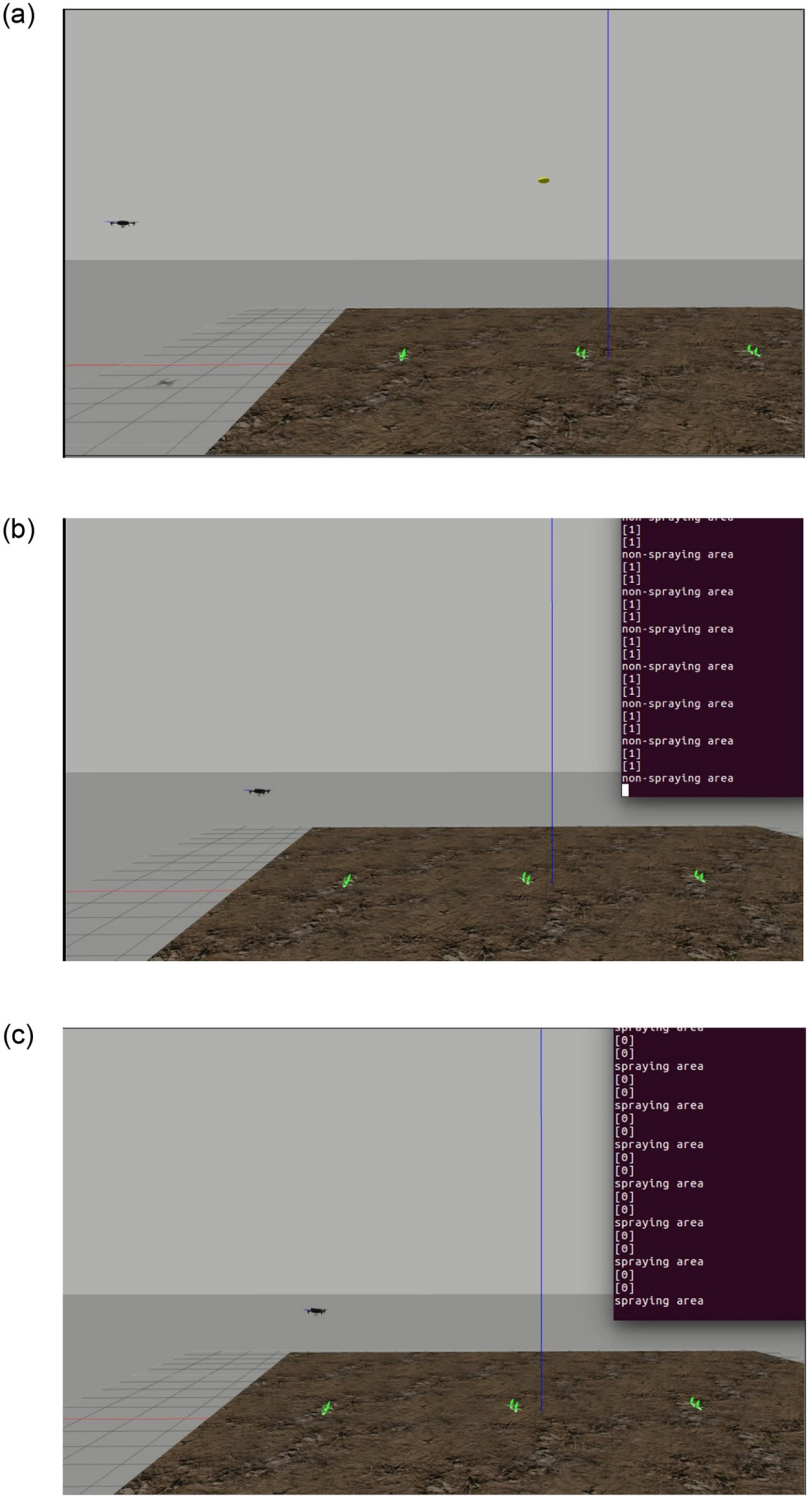
Simulation environment, UAV moving from one-way point to another while searching for the target a) UAV taking off b) searching target c) target recognized.

Fig 5a shows the UAV taking off and moving from one waypoint to another. Fig 5b depicts UAV searching for a target, while Fig 5c shows target recognized in real-time using the tensor flow at the backend.

A custom data set consisting of targets (spraying area), and non-targets (non-spraying area) was established to evaluate the developed system. A total of 10000 images for the spraying class and 6000 for the non-spraying area were collected. 70% of data was deployed for training, while 15% was used for validation and testing.

### Field experiment

**Fig 6:**
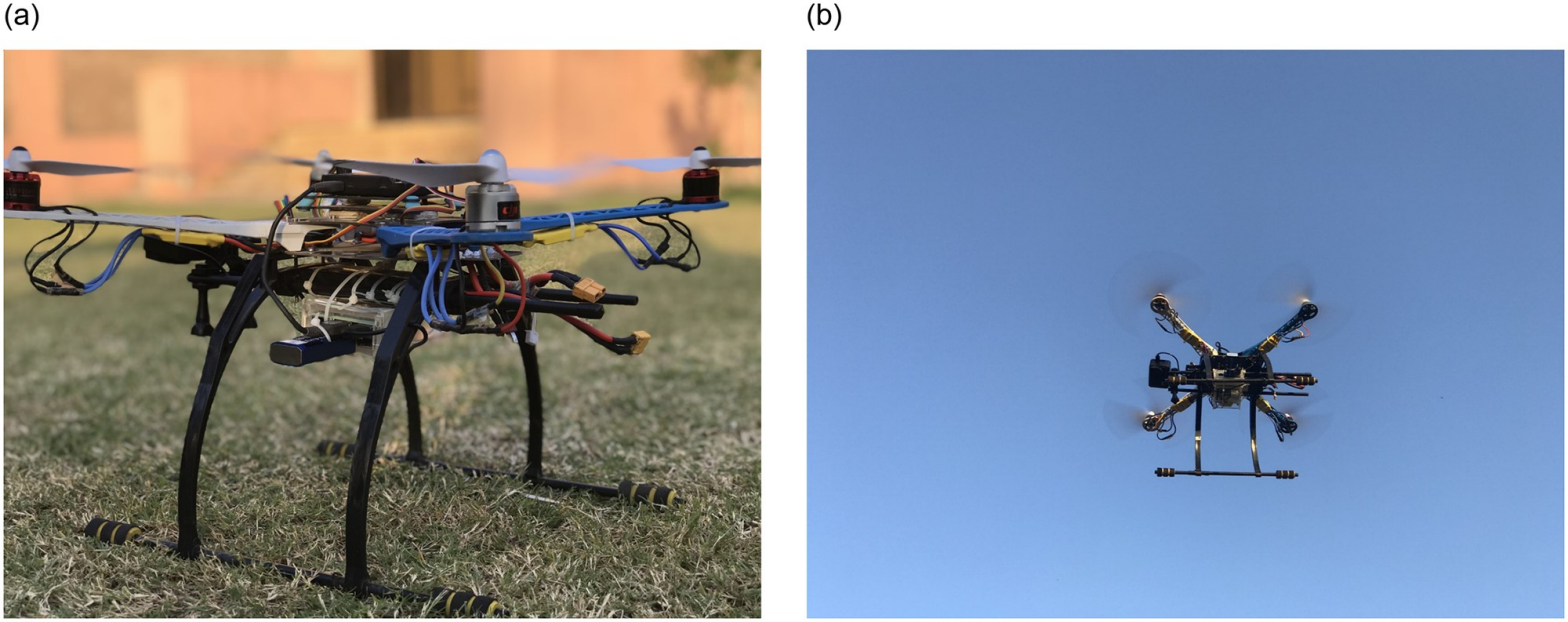
UAV used in the experiment.

Flight tests were conducted at Turangzai (District Peshawar, Khyber-Pakhtunkhwa, Pakistan, Coordinates 34° ^’^12^’ "^57^"^ North, 71° ^’^44^’ "^50^"^ East) on different days over one month. UAV data for coriander was used in the study. A quadcopter UAV was developed for performing outdoor experiments using an Arducopter open-source autopilot. Fig 6 shows the hardware system deployed in the study for conducting the field experiments. Raspberry Pi4 onboard computer, camera, and intel neural computer stick 2 were attached to the UAV. To acquire images for training a height of 2 meters was selected. The developed framework was executed entirely in the onboard Raspberry Pi4 computer with intel neural computer stick 2.

Two classifier datasets for coriander were collected for the off-board recognition system: one data set for spraying areas and another for non-spraying areas. Images were obtained from the video recorded at the preprocessing stage. A total of 1200 images for the spraying class and 900 for the non-spraying area were obtained. Similar to the simulation experiments, the data were classified into training (70%), validation (15%), and testing (15%). An input image of size 448*448 obtained through preprocessing was given as input images. Images were collected during different lighting conditions in one-month; average temperature and ambient humidity were 20.5°C and 60%, respectively.

## Results

**Fig 7:**
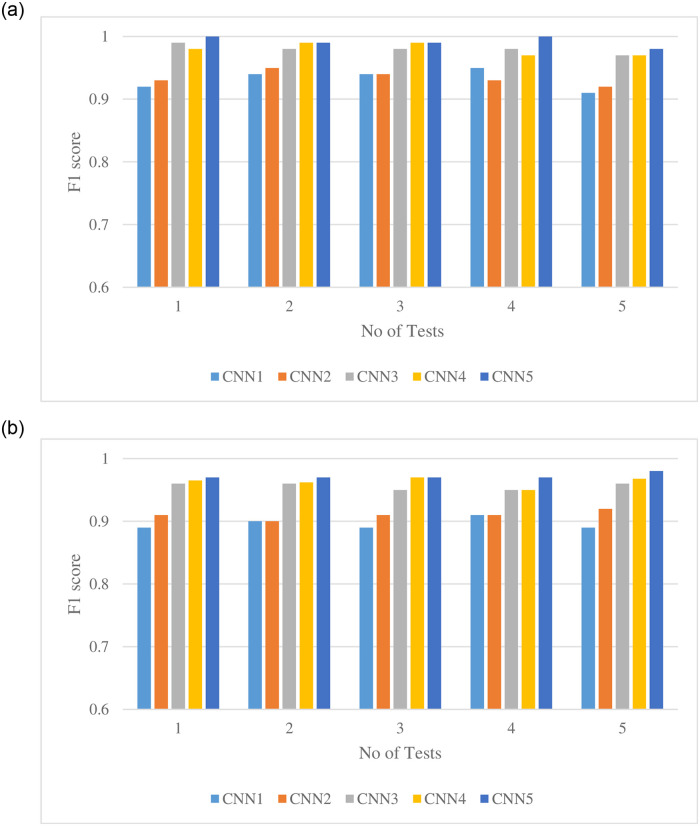
Comparing the supervised learning classifiers’ results on five different evaluation sets a) F1 score for training sets b) F1 score for testing sets.

**Fig 8:**
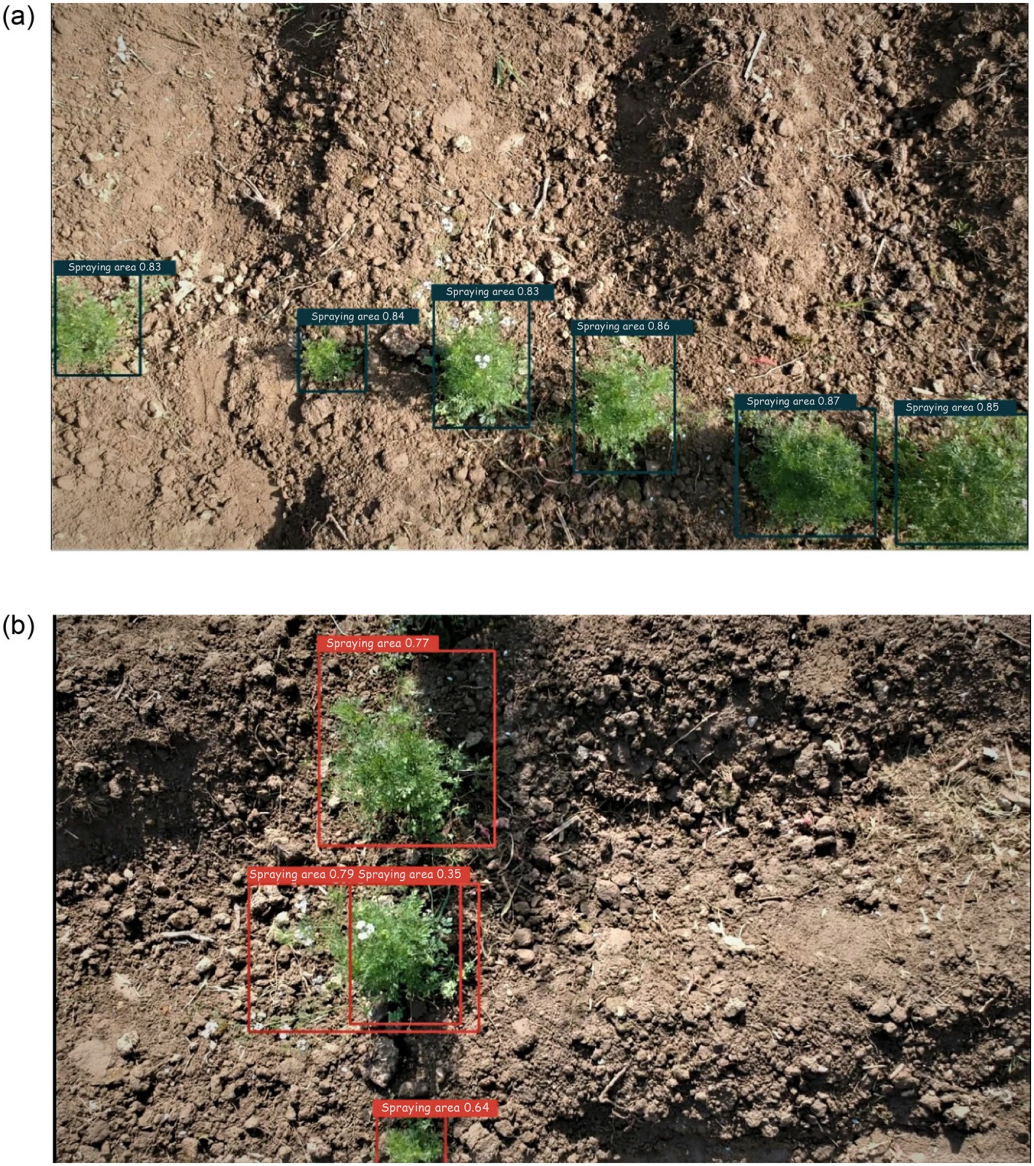
Recognition with a confidence score.

Following rigorous experimentation, encouraging results were recorded for all the classifiers after conducting multiple tests. The results illustrated in Fig 7a and 7b are summarized in Tables 2 and 3, respectively. Average F1 score values of the five tests for training and testing sets are depicted in the following tables. Among the classifiers, CNN5 achieved the best average F1 score of 0.965, followed by CNN4, achieving an average of 0.961. The remaining classifiers (CNN 1, CNN2, and CNN3) achieved an average F1 score of 0.895, 0.915, and 0.955, respectively. The recognition of the spraying area in coriander and their respective confidence score is illustrated in Fig 8.

**Table 2:** Average training results of classifiers for the five evaluation tests.

Target	CNN 1		CNN 2		CNN 3		CNN 4		CNN 5	
	F1 Score	Train Time (s/epoch)	F1 Score	Train Time (s/epoch)	F1 Score	Train Time (s/epoch)	F1 Score	Train Time (s/epoch)	F1 Score	Train Time (s/epoch)
Crops	0.93	28	0.94	29	0.97	30	0.97	33	0.98	35
Non-Crops	0.94		0.95		0.98		0.98		0.99	

**Table 3:** Average testing results of classifiers for the five evaluation tests.

Target	CNN 1		CNN 2		CNN 3		CNN 4		CNN 5	
	F1 Score	Test Time (ms /image)	F1 Score	Test Time (ms /image)	F1 Score	Test Time (ms /image)	F1 Score	Test Time (ms /image)	F1 Score	Test Time (ms /image)
Crops	0.89	3.5	0.91	3.6	0.95	3.68	0.958	4.4	0.96	4.65
Non-Crops	0.90		0.92		0.96		0.964		0.97	

**Fig 9:**
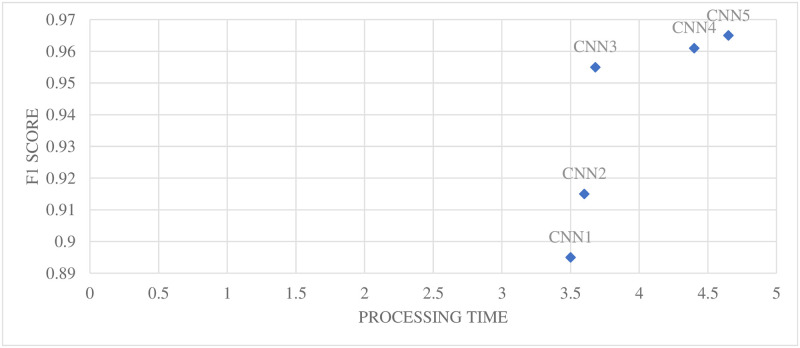
Average F1 score and processing time for all configurations.

Typically, UAVs have limited computational capabilities. So, it is essential to find the balance between the performance and computational cost. Keeping in view this constraint, in addition to the performance (F1 score) of the classifier, test time (processing) is also of great importance (shown in Table 3) while selecting the appropriate classifier for the desired recognition task. The recognition time has been measured through ’UAV’s onboard computer for calculating the processing time per image (average of 256 images). It takes into account the time required for feature extracting and then the desired classification. For better visualization, both the F1 score and test time is plotted in Fig 9.

The best possible classifier taking into account the F1 score (maximizing) and processing time (minimizing), CNN3 has been selected as the most appropriate classifier for the developed method.

### A comparison for evaluation

To justify the capability of the developed method, it was essential to perform a comparative analysis with MSM [31] and two pre-trained models LeNet-5 [35] and VGG 16 [36].

**Fig 10:**
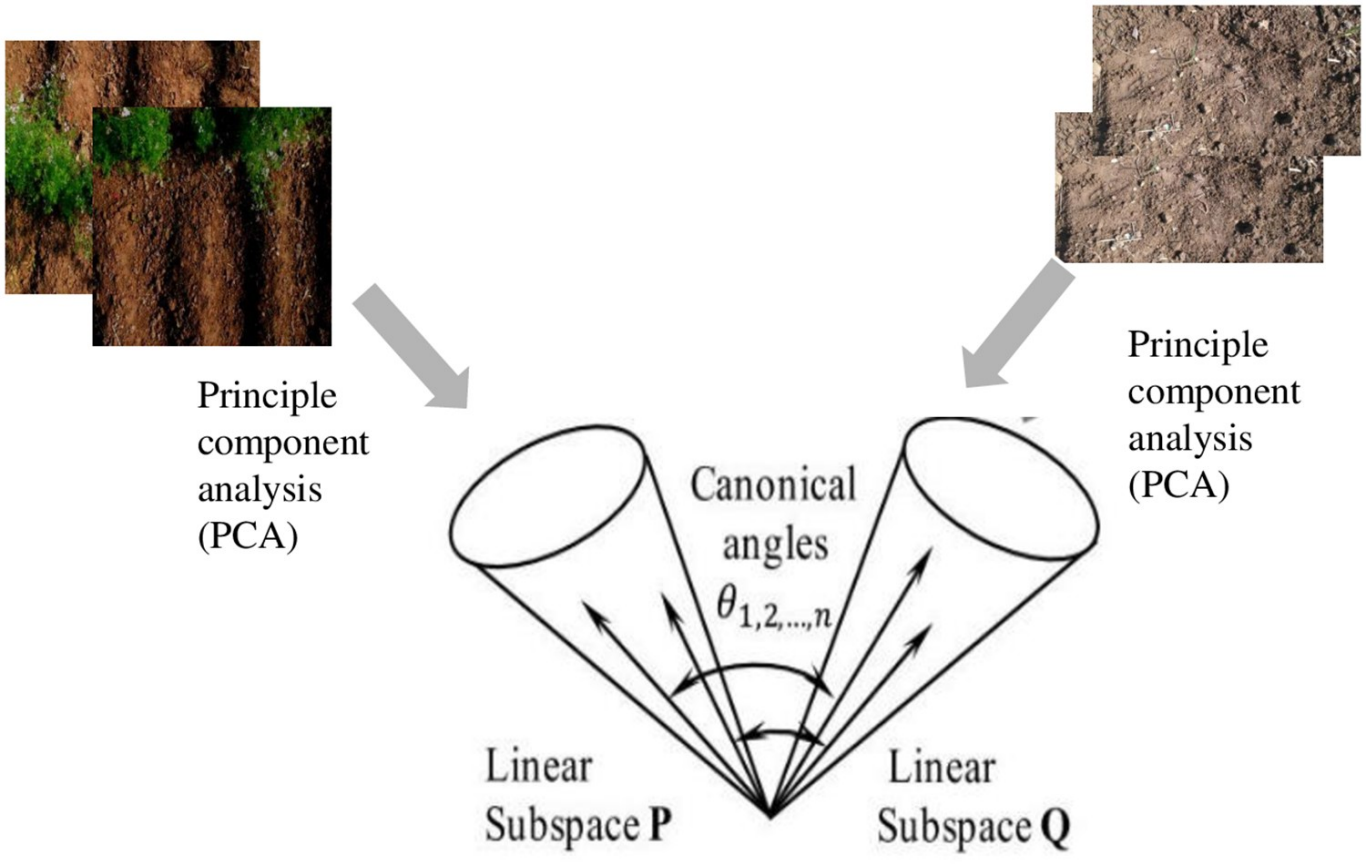
MSM for comparing two sets of images [31].

MSM is usually deployed for recognizing targets based on image sets. It is an extension to the subspace method (SM) by classifying input patterns into their subsequent classes based on multiple canonical angles between the input and class subspaces. The entire process for recognizing targets using MSM is similar to SM except having an input subspace replacing the input vector used in SM [31]. The similarity between the subspace is illustrated in Fig 10.

**Fig 11:**
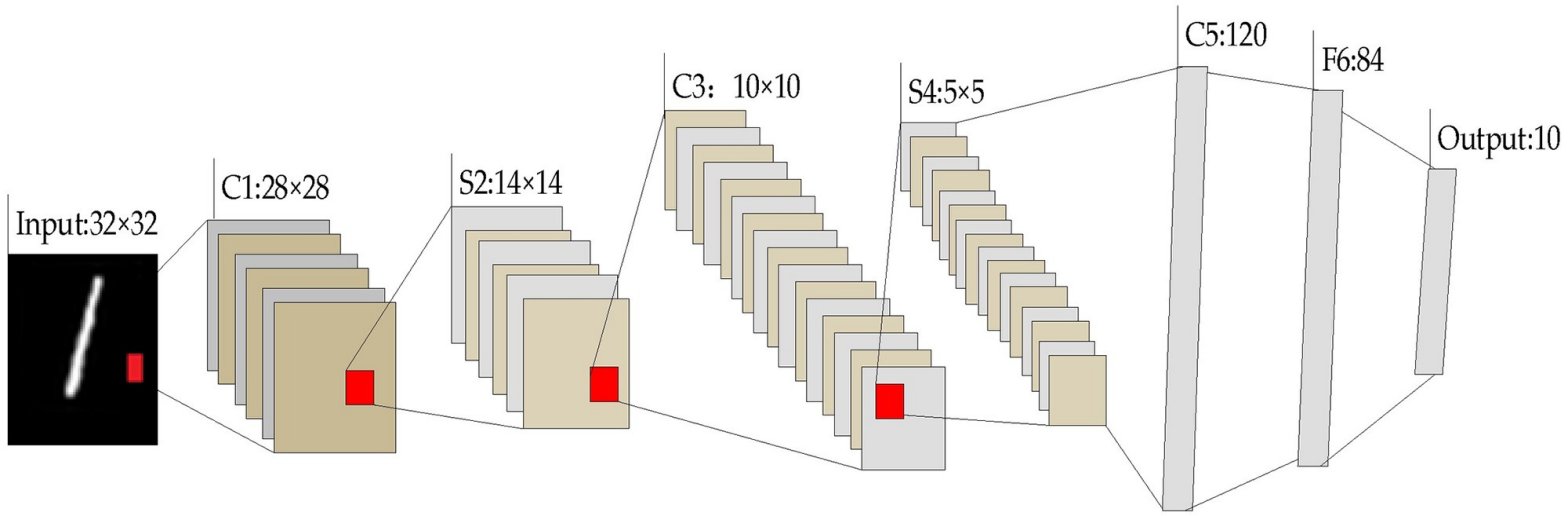
Typical LeNet-5 architecture [35].

LeNet-5 is a classical CNN model developed by Yann Le Cun *et al*. for optical character recognition [35, 37]. A typical LeNet-5 architecture is illustrated in Fig 11. The architecture consists of six layers, comprising three convolutional layers and two sets of pooling layers, and one fully connected layer [35]. The SoftMax classifier is deployed at the end of the model.

**Fig 12:**
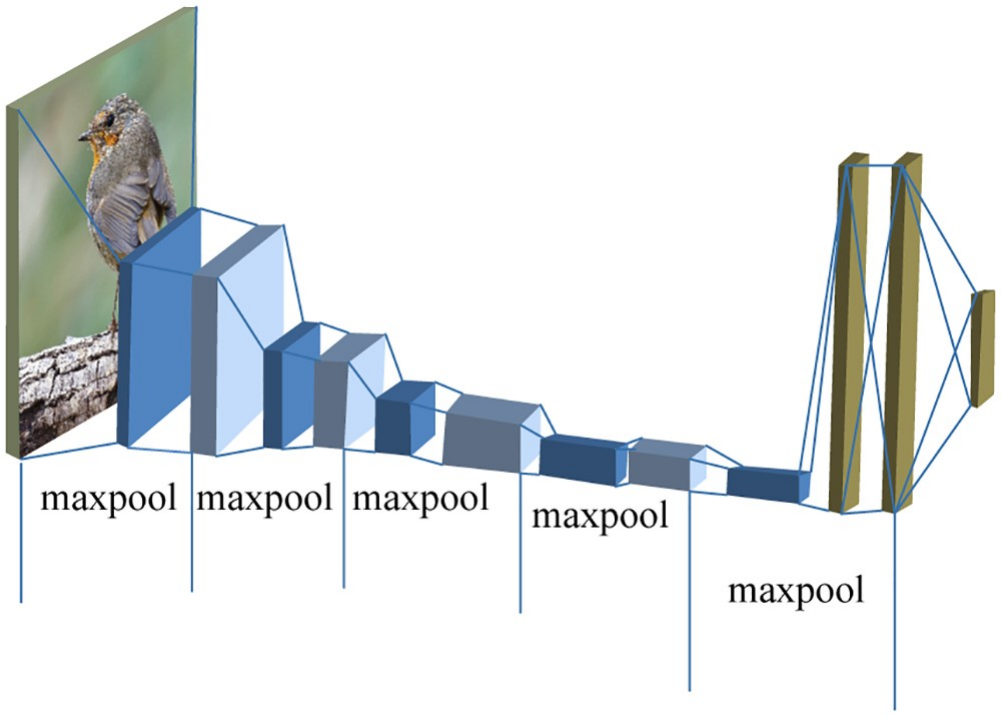
VGG-16 model for bird species classification [36].

The effect of convolutional depth on accuracy in image recognition problem was studied by Simonyan and Zisserman in 2014 and led to the introduction of a new model named Visual Geometry Group (VGG) [36, 37]. One of this group’s special architecture includes VGG- 16, which was used for recognizing handwritten Bengali characters [38]. The architecture of VGG-16 constitutes 13 convolutional layers and three fully connected layers, and a single SoftMax layer [36]. The typical architecture of VGG-16 is illustrated as follows in Fig 12.

The aforementioned models were applied to the same data set, and the average F1 score values obtained for the five tests for testing sets are shown in Table 4.

**Table 4:** Average testing results of classifiers for the 5 evaluation tests.

Target	MSM [31]		LeNet-5 [35]		VGG-16 [36]	
	F1 Score	Test Time (ms /image)	F1 Score	Test Time (ms /image)	F1 Score	Test Time (ms /image)
Crops	0.79	2.9	0.90	3.45	0.97	5.84
Non-Crops	0.80		0.91		0.98	

**Fig 13:**
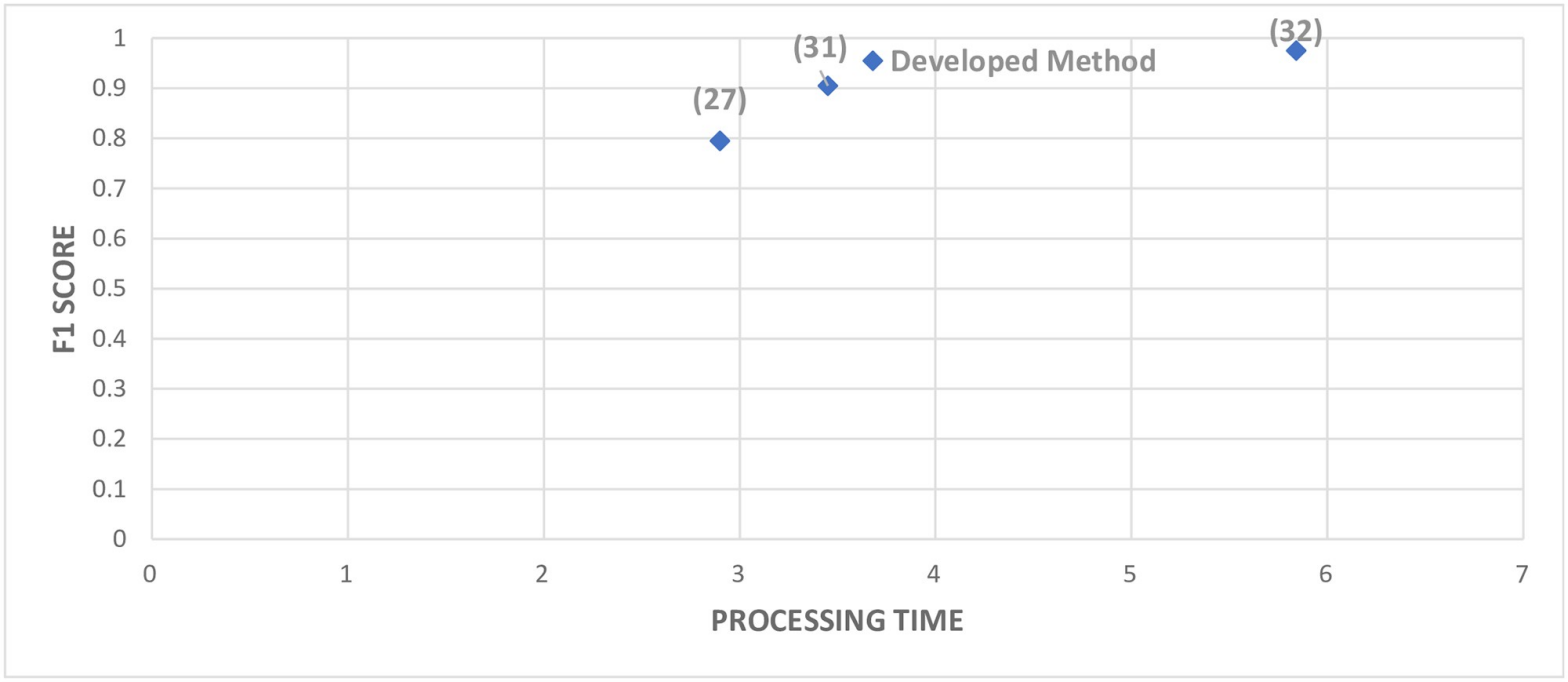
Average F1 score and processing time for comparison.

The overall F1 score of MSM [31] was 0.795 with a recognition time of 2.9ms. Similarly, for LeNet-5 [35], the overall F1 score was 0.905, and recognition time was 3.45ms. Furthermore, the recognition system for VGG-16 achieved an overall F1 score of 0.975 and a time of 5.84ms. A comparison of the models with the selected model is illustrated in Fig 13.

It is evident from Fig 13 that based on the F1 score, the developed model showed improvement compared to MSM (16% increase) and LeNet-5 (5% increase). At the same time, VGG-16 achieved a higher score (2% increase) than the developed model. However, the processing time, which is an essential parameter for the UAV (limited computational capability) in VGG-16, is also higher. So, keeping in view both the parameters, i.e., maximizing the F1 score and minimizing the processing time, the developed model acts as an optimum model for the desired task of recognizing spraying areas in real-time.

## Discussion

It is essential for a country with an agricultural economy like Pakistan to use modern technologies to cope with the emerging challenges to internal food security and achieve international price competitiveness. Millions of dollars are lost due to crop loss and expenses on pesticides. The introduction of agriculture robots is considered a significant boost in this field due to the use of different sensors, electronic and knowledge systems, allowing more precise and cost-efficient monitoring and control of various fluxes on the farm and easier dissemination of information to the farmers. In this regard, a robust real-time spraying area recognition system for UAV based sprayer was developed. A simulated environment similar to the real-world experiment was employed for refining the system, while the coriander field was selected for performing field experiments and testing the system. The data were collected on different days over a month, and our system yielded efficient results, as evident from the data set. UAVs usually operate at high speed and have limited battery life. As a result, it requires high computational speed and fast operation with optimal recognition capability. Given these constraints, the developed real-time recognition system achieves considerably high accuracy with less processing time, which is essential for achieving the desired task.

The developed deep learning system was compared with existing methods to prove its efficacy. Based on testing results and ground truth information, it was observed that the developed system was able to achieve better results. Though VGG 16 achieved higher accuracy than the developed method, it also had higher processing time, which is an important parameter when working with UAV as it has limited computational capability. Thus, making the developed model an ideal system for the study. The developed deep learning system can easily recognize targets with minimal processing time and can be easily incorporated into different precision agriculture applications such as recognizing pests/bugs, weed control, yield estimation, crop health monitoring, etc. The developed system has the potential to be deployed on UAVs for the aforementioned precision agriculture applications.

## Conclusion

In this study, a deep learning-based real-time recognition system was developed for a UAV. The system was based on a flexible architecture that can perform real-time recognition in a fully unsupervised manner. This system’s capability was achieved through a two-step process, where the target recognizer component is based on a CNN model. Different supervised learning classifiers were extensively assessed for the desired target recognition purpose. The final selected model consists of five (05) convolutional layers, four (04) max-pooling layers, five (05) dropout layers, two (02) dense layers with one (01) fully connected layer having a hidden layer of 512 units, and the last layer is a dense layer with sigmoid activation. The developed system was compared with the existing methods, and on the comparison, our model was able to perform better than machine learning (MSM) and current pre-trained deep learning models (LeNet-5, VGG16) based on the two essential parameters, i.e., accuracy and processing time. The developed system achieved an F1 score of 0.955 with a processing time of 3.68 ms. It showed a good tradeoff between accuracy and computational cost, addressing the hard-computational constraint associated with a UAV. The integration of the real-time recognition system into an autonomous UAV spraying system is in progress as part of our future endeavors.

## References

[1] Pakistan Bureau of Statistics, “Agriculture Statistics,” 2019. [Online]. http://www.pbs.gov.pk/content/agriculture-statistics. [Accessed: 10-Nov-2020].

[2] Ministry of Finance, “Agriculture.” [Online]. http://www.finance.gov.pk/survey/chapter_10/02_agriculture.pdf. [Accessed: 10-Nov-2020].

[3] Mogili U. M. R. and Deepak B. B. V. L., “ScienceDirect ScienceDirect Review on Application of Drone Systems in Precision Agriculture,” in *Procedia Computer Science*, 2018, vol. 133, pp. 502–509, 10.1016/j.procs.2018.07.063

[4] Huang Y. B., Thomson S. J., Hoffmann W. C., Bin Lan Y., and Fritz B. K., “Development and prospect of unmanned aerial vehicle technologies for agricultural production management,” *Int*. *J*. *Agric*. *Biol*. *Eng*., vol. 6, no. 3, pp. 1–10, 2013, 10.3965/j.ijabe.20130603.001

[5] Faiçal B. S. et al., “An adaptive approach for UAV-based pesticide spraying in dynamic environments,” vol. 138, pp. 210–223, 2017, 10.1016/j.compag.2017.04.011

[6] Alexandridis T. K. et al., “Novelty detection classifiers in weed mapping: Silybum marianum detection on UAV multispectral images,” *Sensors (Switzerland)*, vol. 17, no. 9, 2017, 10.3390/s17092007 28862663 PMC5621143

[7] Valente J., Doldersum M., Roers C., and Kooistra L., “DETECTING RUMEX OBTUSIFOLIUS WEED PLANTS in GRASSLANDS from UAV RGB IMAGERY USING DEEP LEARNING,” *ISPRS Ann*. *Photogramm*. *Remote Sens*. *Spat*. *Inf*. *Sci*., vol. 4, no. 2/W5, pp. 179–185, 2019, 10.5194/isprs-annals-IV-2-W5-179-2019

[8] Pérez-Ortiz M., Peña J. M., Gutiérrez P. A., Torres-Sánchez J., Hervás-Martínez C., and López-Granados F., “A semi-supervised system for weed mapping in sunflower crops using unmanned aerial vehicles and a crop row detection method,” *Appl*. *Soft Comput*. *J*., vol. 37, pp. 533–544, 2015, 10.1016/j.asoc.2015.08.027

[9] Albetis J. et al., “On the potentiality of UAV multispectral imagery to detect Flavescence dorée and Grapevine Trunk Diseases,” *Remote Sens*., vol. 11, no. 1, 2019, 10.3390/rs11010023

[10] Abdulridha J., Batuman O., and Ampatzidis Y., “UAV-based remote sensing technique to detect citrus canker disease utilizing hyperspectral imaging and machine learning,” *Remote Sens*., vol. 11, no. 11, 2019, 10.3390/rs11111373

[11] Su J. et al., “Wheat yellow rust monitoring by learning from multispectral UAV aerial imagery,” *Comput*. *Electron*. *Agric*., vol. 155, no. August, pp. 157–166, 2018, 10.1016/j.compag.2018.10.017

[12] Lv Z., “The security of Internet of drones,” *Comput*. *Commun*., vol. 148, no. August, pp. 208–214, 2019, 10.1016/j.comcom.2019.09.018

[13] Lv Z., Qiao L., Li J., and Song H., “Deep learning enabled security issues in the Internet of Things,” *IEEE Internet Things J*., 2020.

[14] Lv Z., Yang H. A. N., Singh A. K., Manogaran G., and Lv H., “Trustworthiness in Industrial IoT Systems Based on Artificial Intelligence,” *IEEE Trans*. *Ind*. *Informatics*, 2020.

[15] Lv Z., Zhang S., and Xiu W., “Solving the Security Problem of Intelligent Transportation System With Deep Learning,” *IEEE Trans*. *Intell*. *Transp*. *Syst*., 2020.

[16] Sampedro C., Rodriguez-Ramos A., Bavle H., Carrio A., de la Puente P., and Campoy P., “A Fully-Autonomous Aerial Robot for Search and Rescue Applications in Indoor Environments using Learning-Based Techniques,” *J*. *Intell*. *Robot*. *Syst*. *Theory Appl*., pp. 1–27, 2018, 10.1007/s10846-018-0898-1

[17] Hinas A., Ragel R., Roberts J., and Gonzalez F., “A Framework for Vision-Based Multiple Target Finding and Action Using Multirotor UAVs,” *Sensors*, no. 1, pp. 1320–1327, 2020, 10.1109/ICUAS.2018.8453313 PMC6982733 31947777

[18] Puri V., Nayyar A., and Raja L., “Agriculture drones: A modern breakthrough in precision agriculture,” *J*. *Stat*. *Manag*. *Syst*., vol. 20, no. 4, pp. 507–518, 2017, 10.1080/09720510.2017.1395171

[19] Huang Y., Hoffmann W. C., Lan Y., Wu W., and Fritz B. K., “Development of a spray system for an UAV platform,” vol. 25, no. 6, pp. 803–810, 2009.

[20] Zhu H. et al., “Development of a PWM precision spraying controller for unmanned aerial vehicles,” *J*. *Bionic Eng*., vol. 7, no. 3, pp. 276–283, 2010, 10.1016/S1672-6529(10)60251-X

[21] Huang Y., Hoffman W. C., Lan Y., Fritz B. K., and Thomson S. J., “Development of a Low-Volume Sprayer for an Unmanned Helicopter,” *J*. *Agric*. *Sci*., vol. 7, no. 1, pp. 148–153, 2014, 10.5539/jas.v7n1p148

[22] Faiçal B. S. et al., “The use of unmanned aerial vehicles and wireless sensor networks for spraying pesticides,” *J*. *Syst*. *Archit*., vol. 60, no. 2014, pp. 393–404, 2014, 10.1016/j.sysarc.2014.01.004

[23] Guidetti R., Bodria L., Best S., Giles D. K., and Billing R. C., “Deployment and Performance of a UAV for Crop Spraying,” *Chem*. *Eng*. *Trans*., vol. 44, pp. 307–312, 2015, 10.3303/CET1544052

[24] Faiçal B. S., Pessin G., Filho G. P. R., Carvalho A. C. P. L. F., Gomes P. H., and Ueyama J., “Fine-Tuning of UAV Control Rules for Spraying Pesticides on Crop Fields: An Approach for Dynamic Environments,” *Int*. *J*. *Artif*. *Intell*. *Tools*, vol. 25, no. 1, pp. 1–19, 2016, 10.1142/S0218213016600034

[25] Xue X., Lan Y., Sun Z., Chang C., and Hoffmann W. C., “Develop an unmanned aerial vehicle based automatic aerial spraying system,” *Comput*. *Electron*. *Agric*., vol. 128, pp. 58–66, 2016, 10.1016/j.compag.2016.07.022

[26] Spoorthi.S Dr.B.Shadaksharappa Suraj.S and V.K.Manasa, “Freyr drone: Pesticide/ fertilizers spraying drone,” in IEEE 2nd International Conference on In Computing and Communications Technologies, 2017, vol. 3 pages, no. 2017, pp. 252–255.

[27] V. P. Yallappa D, M. Veerangouda, Devanand Maski, “DEVELOPMENT AND EVALUATION OF DRONE MOUNTED SPRAYER FOR PESTICIDE APPLICATIONS TO CROP,” in IEEE Global Humanitarian Technology Conference, 2015.

[28] B. Dai, Y. He, F. Gu, L. Yang, J. Han, and W. Xu, “A vision-based autonomous aerial spray system for precision agriculture,” 2017 IEEE Int. Conf. Robot. Biomimetics, ROBIO 2017, vol. 2018-Janua, pp. 1–7, 2018.

[29] Wen S., Zhang Q., Yin X., Lan Y., and Zhang J., “Design of Plant Protection UAV Variable Spray,” *Sensors (Switzerland)*, vol. 19, no. 1, 2019, 10.3390/s19051112 30841563 PMC6427117

[30] Pimentel D. and Burgess M., “Small amounts of pesticides reaching target insects,” *Environ*. *Dev*. *Sustain*., vol. 14, no. 1, pp. 1–2, 2012, 10.1007/s10668-011-9325-5

[31] Gao P., Zhang Y., Zhang L., Noguchi R., and Ahamed T., “Article development of a recognition system for spraying areas from unmanned aerial vehicles using a machine learning approach,” *Sensors (Switzerland)*, vol. 19, no. 2, 2019, 10.3390/s19020313 30646586 PMC6359728

[32] Huang H., Deng J., Lan Y., Yang A., Deng X., and Zhang L., “A fully convolutional network for weed mapping of unmanned aerial vehicle (UAV) imagery,” *PLoS One*, vol. 13, no. 4, 2018, 10.1371/journal.pone.0196302 29698500 PMC5919481

[33] Khan S., Tufail M., and Khan M. T., “Deep learning based spraying area recognition system for Unmanned Aerial Vehicle based sprayers,” *Turkish J*. *Electr*. *Eng*. *Comput*. *Sci*., vol. 29, no. 2021, pp. 241–256, 2021, 10.3906/elk-2004-4

[34] “Gazebo.” [Online]. http://gazebosim.org/. [Accessed: 22-Nov-2020].

[35] Wei G., Li G., Zhao J., and He A., “Development of a LeNet-5 gas identification CNN structure for electronic noses,” *Sensors (Switzerland)*, vol. 19, no. 1, 2019, 10.3390/s19010217 30626158 PMC6339057

[36] Islam S., Khan S. I. A., Minhazul Abedin M., Habibullah K. M., and Das A. K., “Bird species classification from an image using VGG-16 network,” *ACM Int*. *Conf*. *Proceeding Ser*., pp. 38–42, 2019, 10.1145/3348445.3348480

[37] Rahman M. M., Islam M. S., Sassi R., and Aktaruzzaman M., “Convolutional neural networks performance comparison for handwritten Bengali numerals recognition,” *SN Appl*. *Sci*., vol. 1, no. 12, pp. 1–11, 2019, 10.1007/s42452-019-1682-y

[38] Alom M. Z., Sidike P., Hasan M., Taha T. M., and Asari V. K., “Handwritten bangla character recognition using the state-of-art deep convolutional neural networks,” *arXiv*, pp. 1–12, 2017. 10.1155/2018/6747098 PMC6129853 30224913

