# Expression of Concern: Real-time recognition of spraying area for UAV sprayers using a deep learning approach

**DOI:** 10.1371/journal.pone.0326610

**Published:** 2025-06-20

**Authors:** The PLOS One Editors

The *PLOS One* Editors issue this Expression of Concern to inform readers that concerns about aspects of the article’s peer review were identified which may have implications for the reliability of research reported in this article. Readers are advised to interpret this article [1] with caution.

In addition, references 12–15 and the corresponding cited statement in [1] were added during revision of the manuscript but do not correspond to requested revisions in the reviews or decision letter and do not appear to be fully relevant in the cited context. The corresponding author confirmed that the addition of these references was not requested by reviewers or the Editorial Board member who handled peer review, and asserted that the added citations are pertinent to the topic of this article [1].
